# The Connected *P*-Median Problem on Cactus Graphs

**DOI:** 10.1155/2021/3533623

**Published:** 2021-12-28

**Authors:** Chunsong Bai, Jianjie Zhou, Zuosong Liang

**Affiliations:** ^1^School of Finance and Mathematics, Huainan Normal University, Huainan 232038, China; ^2^School of Information and Management Science, Henan Agricultural University, Zhengzhou 450002, China; ^3^School of Management, Qufu Normal University, Rizhao 276800, China

## Abstract

This study deals with the facility location problem of locating a set *V*_*p*_ of *p* facilities on a graph such that the subgraph induced by *V*_*p*_ is connected. We consider the connected *p*-median problem on a cactus graph *G* whose vertices and edges have nonnegative weights. The aim of a connected *p*-median problem is to minimize the sum of weighted distances from every vertex of a graph to the nearest vertex in *V*_*p*_. We provide an *O*(*n*^2^*p*^2^) time algorithm for the connected *p*-median problem, where *n* is the number of vertices.

## 1. Introduction

The *p*-median and *p*-center problems are central to the field of location theory and logistics and are now well studied in operations research. Applications of the two problems include the location of industrial plants, warehouses, distribution centers, and public service facilities in transportation networks, as well as the location of various facilities in telecommunication networks [1, 2].

To determine the backup sites or to balance the workloads among the center vertices in real networks effectively, Yen and Chen [3] originally proposed the connected *p*-center problem and showed that the connected *p*-center problem is NP-hard on both bipartite graphs and split graphs. Yen [4] studied the connected *p*-center problem on block graphs. Bai et al. [5, 6] considered the connected *p*-center problem on cactus graphs and devised an *O*(*n*^2^*p*^2^) algorithm.

Similar to the connected *p*-center problem, Shan et al. [7] introduced the connected *p*-median problem and considered the connected *p*-median problems on interval and circular-arc graphs. Kang et al. [8] studied the connected *p*-median problem on block graphs and proved that the problem is linearly solvable on block graphs which have unit edge lengths.

In this study, we consider the problem of finding the optimal location of connected *p*-median on a cactus graph. The study is organized as follows. In the next section, we formally introduce the notations and the problem that we studied in this study. In Section 3, we study the connected *p*-median problem on a cactus graph and devise an algorithm with time complexity of *O*(*n*^2^*p*^2^). Finally, we conclude this study.

## 2. Problem Formulation

Let *G*=(*V*, *E*, *w*, *l*) be a connected graph with vertex set *V*(|*V*|=*n*) and edge set *E*(|*V*|=*n*), where each vertex *v* ∈ *V* (or *v*_*i*_ ∈ *V*) has a weight *w*(*v*) ≥ 0 (or *w*_*i*_ ≥ 0) and each edge *e* ∈ *E* has a certain length *l*(*e*). For any two vertices *u*, *v*, let *P*[*u*, *v*] be the shortest path from *u* to *v*, and *d*(*u*, *v*) be the length of *P*[*u*, *v*]. A *p*-vertex set *X*_*p*_ is called a connected *p*-vertex set if the induced subgraph of *X*_*p*_ is connected.

A cycle is a sequence (*v*_1_,…, *v*_*s*_, *v*_*s*+1_=*v*_1_) of *s*(*s* ≥ 3) clockwise indexed vertices, such that (*v*_*i*_, *v*_*i*+1_) is an edge, 1 ≤ *i* ≤ *s*. A graph *G* is called a cactus graph if any two cycles of *G* have at most one vertex in common. The vertex set of a cactus graph *G* can be divided into three disjoint subsets: *G*-vertices, *C*-vertices, and hinges. A vertex is called a *C*-vertex if it is in a cycle of *G* and its degree is 2 in *G*. A vertex is called a hinge if it is in a cycle of *G* and its degree is at least three. A vertex is called a *G*-vertex if it is not in a cycle of *G*.

Given a cactus graph *G*, a subtree is a connected subgraph of *G* induced by some *G*-vertices and hinges that does not contain any cycle. A subtree is called as a graft if it is maximal and without two hinges belonging to the same cycle. A cycle or a graft is called a block of *G*.

**Figure 1:**
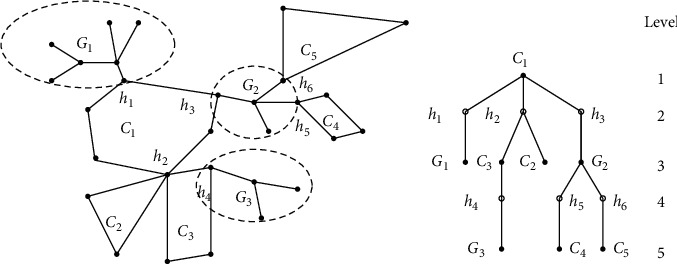
(a) A cactus graph *G* with five cycles *C*_1_,…, *C*_5_, three grafts *G*_1_, *G*_2_, *G*_3_, and six hinges *h*_1_,…, *h*_6_. (b) The tree structure *T*_*G*_ of *G*.

For convenience, we use a tree *T*_*G*_ to represent the skeleton of *G* (Figure 1). Then, we convert *T*_*G*_ into a rooted tree as follows: select an arbitrary block, e.g., *B*_0_, as the “root” of *T*_*G*_. For each vertex (block or hinge) *v* in *T*_*G*_, let lev(*v*) be the level of *v*. Let *L*_*m*_=max_*v*∈*T*_*G*__{lev(*v*)} be the maximal one of all levels. For each hinge *h*, by deleting the last edge of the path from *B*_0_ to *h*, we obtain two subtrees of *T*_*G*_. Let *G*_*h*_ be the subcacti of *G* induced by the vertices of *h* and all subcacti hanging from it, and let *G*_*h*_^*c*^ be the subcacti *G* − *G*_*h*_. Note that the father of any block is always a hinge, which is called the block's companion hinge. For simplicity, we choose an arbitrary vertex *h*_0_ ∈ *B*_0_ as the virtual companion hinge of *B*_0_. Denote by *B*_*h*_ the block *B* if its companion hinge is *h*. Let *G*_*B*,*h*_ be the subcactus of *G* induced by the vertices of *B*_*h*_ and all subcacti hanging from *B*_*h*_, and let *G*_*B*,*h*_^*c*^ be the subcacti *G* − *G*_*B*,*h*_. Specially, *G*=*G*_*h*_0__=*G*_*B*_0_,*h*_0__ and *G*_*h*_0__^*c*^=*G*_*B*_0_,*h*_0__^*c*^=∅.

For each *k*-vertex set *X*_*k*_ in *G*, let *F*_*G*_(*X*_*k*_) be the sum of weighted distances from all vertices in *G* to *X*_*k*_, that is,

(1) $$\begin{array}{c} F_{G}\left(X_{k}\right)=\sum_{v\in V\left(G\right)}w\left(v\right)d\left(v,X_{k}\right), \end{array}$$

where *d*(*v*, *X*_*k*_)=min_*x*_*i*_∈*X*_*k*__*d*(*v*, *x*_*i*_).

**Problem 1 .**

Find a connected *p*-vertex set *X*_*p*_ in *G*, such that *F*_*G*_(*X*_*p*_) is minimized. This problem is known as the connected *p*-median problem (CpM). The optimal solution *X*_*p*_^*∗*^ is called a connected *p*-median of *G*.

Given a graft *B*_*h*_ of *G* and an integer *k*, 1 ≤ *k* ≤ *p*, for each vertex *v* ∈ *B*_*h*_, the aim of the problem *P*(*G*_*B*,*h*_, *v*, *k*) is to find a connected *k*-vertex set *V*(*G*_*B*,*h*_, *v*, *k*) of *G*_*B*,*h*_, such that *F*_*G*_*B*,*h*__(*V*(*G*_*B*,*h*_, *v*, *k*)) is minimized and *v* is the closest vertex to *h* in *V*(*G*_*B*,*h*_, *v*, *k*)∩*V*(*B*_*h*_). We call the corresponding subset *V*(*G*_*B*,*h*_, *v*, *k*) as *v*-restricted connected *k*-median of *G*_*B*,*h*_.

Given a cycle *B*_*h*_ of *s* indexed vertices *v*_1_=*h*, *v*_2_,…, *v*_*s*_ and an integer *k*, 1 ≤ *k* ≤ *p*, for any two vertices {*v*_*i*_, *v*_*j*_} ⊂ *V*(*B*_*h*_), the aim of the problem *P*(*G*_*B*,*h*_, {*v*_*i*_, *v*_*j*_}, *k*) is to find a connected *k*-vertex set *V*(*G*_*B*,*h*_, {*v*_*i*_, *v*_*j*_}, *k*) of *G*_*B*,*h*_, such that *F*_*G*_*B*,*h*__(*V*(*G*_*B*,*h*_, {*v*_*i*_, *v*_*j*_}, *k*)) is minimized and *V*(*G*_*B*,*h*_, {*v*_*i*_, *v*_*j*_}, *k*)∩*V*(*B*_*h*_) contains only the vertices of the path from *v*_*i*_ to *v*_*j*_ on *B*_*h*_ in clockwise direction. The corresponding subset *V*(*G*_*B*,*h*_, {*v*_*i*_, *v*_*j*_}, *k*) is called as {*v*_*i*_, *v*_*j*_}-restricted clockwise connected *k*-median of *G*_*B*,*h*_.

Given a subgraph *G*_*h*_ and an integer *k*, 1 ≤ *k* ≤ *p* for each hinge of *G*_*h*_, the aim of the problem *P*(*G*_*h*_, *h*, *k*) is to find a connected *k*-vertex set *V*(*G*_*h*_, *h*, *k*) of *G*_*h*_, such that *F*_*G*_*h*__(*V*(*G*_*h*_, *h*, *k*)) is minimized. The corresponding subset *V*(*G*_*h*_, *h*, *k*)*h*-restricted is called as connected *k*-median of *G*_*h*_.

For all subcacti *G*_*B*,*h*_ of *G*, denote by *𝒱*_1_ (respectively, *𝒱*_2_) the possible *v*-restricted ({*v*_*i*_, *v*_*j*_}-restricted clockwise) connected *p*-medians *V*(*G*_*B*,*h*_, *v*, *p*) (respectively, *V*(*G*_*B*,*h*_, {*v*_*i*_, *v*_*j*_}, *p*)). For all hinges *h* of *G*, denote by *𝒱*_3_ the possible *h*-restricted connected *p*-medians *V*(*G*_*h*_, *h*, *p*). The following lemma establishes a significant relationship between the CpM problems on *G*_*h*_0__ and all restricted *p*-median problems.

**Lemma 1 .**

Given a cactus graph *G*_*h*_0__, there exists a connected *p*-median *X*_*p*_ in *𝒱*_1_ ∪ *𝒱*_2_ ∪ *𝒱*_3_.

**Proof**

Suppose that *v* ∈ *X*_*p*_ is the closest vertex to *h*_0_ in *G*. Then, *v* could be a *G*-vertex, a *C*-vertex, or a hinge. We distinguish three cases.

**Case 1 .**

Given a graft *B*_*h*_ of *G* and a *G*-vertex *v* of *B*_*h*_, suppose that *V*(*G*_*B*,*h*_, *v*, *p*) is a *v*-restricted connected *p*-median of *G*_*B*,*h*_, then *V*(*G*_*B*,*h*_, *v*, *p*) is a feasible solution to the CpM problem, that is,

(2) $$\begin{array}{c} F_{G}\left(V\left(G_{B,h},v,p\right)\right)\geq F_{G}\left(X_{p}\right). \end{array}$$

On the other hand, since *X*_*p*_ is also a *v*-restricted connected *p*-vertex of *G*_*B*,*h*_, we have

(3) $$\begin{array}{c} F_{G}\left(X_{p}\right)=F_{G_{B,h}}\left(X_{p}\right)+\sum_{u\in V\left(G_{B,h}^{c}\right)}w\left(u\right)\left[d\left(u,h\right)+d\left(h,v\right)\right]\\ \geq F_{G_{B,h}}\left(V\left(G_{B,h},v,p\right)\right)+\sum_{u\in V\left(G_{B,h}^{c}\right)}w\left(u\right)\left[d\left(u,h\right)+d\left(h,v\right)\right]\\ =F_{G}\left(V\left(G_{B,h},v,p\right)\right). \end{array}$$

Thus, *F*_*G*_(*X*_*p*_)=*F*_*G*_(*V*(*G*_*B*,*h*_, *v*, *p*)), which means *V*(*G*_*B*,*h*_, *v*, *p*) is a connected *p*-median of *G*_*h*_0__.

**Case 2 .**

Given a cycle *B*_*h*_ of *G* and two *C*-vertices *v*_*i*_, *v*_*j*_ of *B*_*h*_, *i* ≤ *j*. Suppose that *X*_*p*_∩*V*(*B*_*h*_) includes only the vertices of the path from *v*_*i*_ to *v*_*j*_ on *B*_*h*_ in clockwise direction, and *V*(*G*_*B*,*h*_, {*v*_*i*_, *v*_*j*_}, *p*) is a {*v*_*i*_, *v*_*j*_}-restricted clockwise connected *p*-median of *G*_*B*,*h*_. Similar as Case 1, we deduce that

(4) $$\begin{array}{c} F_{G}\left(V\left(G_{B,h},\left\{v_{i},v_{j}\right\},p\right)\right)\geq F_{G}\left(X_{p}\right),\\ F_{G}\left(X_{p}\right)=F_{G_{B,h}}\left(X_{p}\right)+\sum_{u\in V\left(G_{B,h}^{c}\right)}w\left(u\right)\left[d\left(u,h\right)+\min\left\{d\left(h,v_{i}\right),d\left(h,v_{j}\right)\right\}\right]\\ \geq F_{G_{B,h}}\left(V\left(G_{B,h},\left\{v_{i},v_{j}\right\},p\right)\right)+\sum_{u\in V\left(G_{B,h}^{c}\right)}w\left(u\right)\left[d\left(u,h\right)+\min\left\{d\left(h,v_{i}\right),d\left(h,v_{j}\right)\right\}\right]\\ =F_{G}\left(V\left(G_{B,h},\left\{v_{i},v_{j}\right\},p\right)\right). \end{array}$$

Thus, *F*_*G*_(*X*_*p*_)=*F*_*G*_(*V*(*G*_*B*,*h*_, {*v*_*i*_, *v*_*j*_}, *p*)), *V*(*G*_*B*,*h*_, {*v*_*i*_, *v*_*j*_}, *p*) is a connected *p*-median of *G*_*h*_0__.

**Case 3 .**

For the case *v*=*h* is a hinge, we distinguish three subcases.

Subcase 3.1. The neighbor of *h* is a graft *B*_*h*_. We can obtain *F*_*G*_(*X*_*p*_)=*F*_*G*_(*V*(*G*_*B*,*h*_, *h*, *p*)) and *V*(*G*_*B*,*h*_, *h*, *p*) is a connected *p*-median of *G*_*h*_0__ through the same discussion as in Case 1.
Subcase 3.2. The neighbor of *h* is a cycle *B*_*h*_, and *X*_*p*_∩*V*(*B*_*h*_) includes the vertices of the path from *v*_*i*_ to *v*_*j*_ on *B*_*h*_ in clockwise direction, *i* ≤ *j*. We can obtain *F*_*G*_(*X*_*p*_)=*F*_*G*_(*V*(*G*_*B*,*h*_, {*v*_*i*_, *v*_*j*_}, *p*)) and *V*(*G*_*B*,*h*_, {*v*_*i*_, *v*_*j*_}, *p*) is a connected *p*-median of *G*_*h*_0__ through the same discussion as in Case 2.
Subcase 3.3. The neighbor of *h* belongs to more than one block. Assume that *V*(*G*_*h*_, *h*, *p*) is a *h*-restricted connected *p*-median of *G*_*h*_. Similar as Case 1, we deduce that
  (5) $$\begin{array}{c} F_{G}\left(V\left(G_{h},h,p\right)\right)\geq F_{G}\left(X_{p}\right),\\ F_{G}\left(X_{p}\right)=F_{G_{h}}\left(X_{p}\right)+\sum_{u\in V\left(G_{h}^{c}\right)}w\left(u\right)d\left(u,h\right)\\ \geq F_{G_{h}}\left(V\left(G_{h},h,p\right)\right)+\sum_{u\in V\left(G_{h}^{c}\right)}w\left(u\right)d\left(u,h\right)\\ =F_{G}\left(V\left(G_{h},h,p\right)\right). \end{array}$$

Thus, *F*_*G*_(*X*_*p*_)=*F*_*G*_(*V*(*G*_*h*_, *h*, *p*)), and *V*(*G*_*h*_, *h*, *p*) is a connected *p*-median of *G*_*h*_0__.

From the discussion above, there exists a vertex-restricted connected *p*-median whose sum of weighted distances of *G*_*h*_0__ is equal to the sum of weighted distances of a connected *p*-median of *G*_*h*_0__.

In view of Lemma 1, we will design an algorithm to find all the restricted connected *p*-medians in *𝒱*_1_ ∪ *𝒱*_2_ ∪ *𝒱*_3_. The algorithm uses the idea of dynamic programming. Traverse the tree *T*_*G*_ “upward,” from the vertices (block or hinge) with higher levels to the vertices with lower levels. An arbitrary order is defined when vertices are with the same levels. In each loop, we select a block *B*_*h*_ or a hinge *h*. If *B*_*h*_ is a graft, we calling program GRAFT (*B*, *h*) to deal with the problem on *G*_*B*,*h*_; otherwise, we call program CYCLE (*B*, *h*). When it comes to *G*_*h*_, we call program HINGE (*G*, *h*). For the further computations, all useful data are transferred to *h*. When the block *B*_*h*_0__ has been checked, these data can be used to find a connected *p*-median *V*_*p*_^*∗*^ of *G*.

## 3. Algorithm for the CpM Problem on Cactus Graph

### 3.1. The Program GRAFT (*B*,*h*)

In this subsection, for each graft *B*_*h*_ of *G* and each possible positive integer *k*, our task is to find all restricted connected *k*-medians *V*(*G*_*B*,*h*_, *v*, *k*).

Denote by *T* the given graft *B*_*h*_. Assume that *T* is rooted at the hinge *h*. Denote by *L*′=max_*v*∈*V*(*T*)_lev(*v*). For each vertex *v* ≠ *h*, we can obtain two subtrees of *T* by deleting the last edge of the path from *h* to *v*. Let *T*_*v*_ be the subtree that contains *v*, and let *T*_*v*_^*c*^=*T* − *T*_*v*_. Let *G*_*B*,*v*_ and *G*_*B*,*h*_^*c*^=*G*_*B*,*h*_ − *G*_*v*_ be the subgraphs of *G*_*h*_ corresponding to *T*_*v*_ and *T*_*v*_^*c*^, respectively.

For each vertex *v* ∈ *T*, let *E*(*v*) be the edges of *T*_*v*_ that are incident with *v*, and let *s*(*v*)=|*E*(*v*)|. After sorting the edges of *E*(*v*) in an arbitrary order, the *l*^th^ edge is denoted by *e*(*v*, *l*). If *e*(*v*, *l*)=(*v*, *v*_*l*_), then *v* is called as the farther far(*v*_*l*_) of *v*_*l*_ and *v*_*l*_ is called as the *l*^th^son of *v*. Let son(*v*) be all sons of *v*. For the subtree *T*_*v*_, let *T*_*e*(*v*, *l*)_ be the maximal connected subtree that contains *v* but not any edge *e*(*v*, *j*) for *j* > *l*. Particularly, *T*_*e*(*v*, 0)_=*v* and *T*_*e*(*v*, *s*(*v*))_=*T*_*v*_.

For the subgraph *G*_*B*,*v*_, let *G*_*B*,*e*(*v*, *l*)_ be the subgraph induced by all vertices of *T*_*e*(*v*, *l*)_ and all subcacti hanging from it. For the subgraph *G*_*B*,*e*(*v*, *l*)_, let *V*(*e*(*v*, *l*), *k*) be the connected *k*-vertex set that contains *v* but not any vertex *v*_*j*_ ∈ son(*v*) for *j* > *l*. We define the sum of weighted distances of *V*(*e*(*v*, *l*), *k*) over *G*_*B*,*e*(*v*, *l*)_ as follows.

**Definition 1 .**

Given a subgraph *G*_*B*,*e*(*v*, *l*)_, the optimal value of *V*(*e*(*v*, *l*), *k*) is defined as

(6) $$\begin{array}{c} f^{*}\left(V\left(e\left(v,l\right),k\right)\right)=\underset{V\left(e\left(v,l\right),k\right)\subseteq G_{B,e\left(v,l\right)}}{\min}f_{G_{B,e\left(v,l\right)}}\left(V\left(e\left(v,l\right),k\right)\right), \end{array}$$

where 1 ≤ *k* ≤ min{*p*, |*G*_*B*,*e*(*v*, *l*)_|}. Let *V*^*∗*^(*e*(*v*, *l*), *k*) be the corresponding set to *f*^*∗*^(*V*(*e*(*v*, *l*), *k*)).

Once we obtain all values *f*^*∗*^(*V*(*e*(*v*, *s*(*v*))), *k*) and *f*_*G*_*B*,*v*_^*c*^_(*v*), the sum of weighted distances from vertices in *G*_*B*,*h*_ to *V*(*G*_*B*,*h*_, *v*, *k*) can be calculated as

(7) $$\begin{array}{c} F_{G_{B,h}}\left(V\left(G_{B,h},v,k\right)\right)=f^{*}\left(V\left(e\left(v,s\left(v\right)\right),k\right)\right)+f_{G_{B,v}^{c}}\left(v\right). \end{array}$$

For the block *B*_*h*_, we first deal with all vertices in leaf(*T*). For each *G*-vertex *v* ∈ leaf(*T*), let

(8) $$\begin{array}{c} f^{*}\left(V\left(e\left(v,0\right),1\right)\right)=0,\\ V^{*}\left(e\left(v,0\right),1\right)=\left\{v\right\}. \end{array}$$

For each hinge vertex *v* ∈ leaf(*T*), let

(9) $$\begin{array}{c} W\left(G_{B,v}\right)=W\left(G_{v}\right),\\ f^{*}\left(V\left(e\left(v,0\right),k\right)\right)=F_{G_{v}}\left(V\left(G_{v},v,k\right)\right),\\ V^{*}\left(e\left(v,0\right),k\right)=V\left(G_{v},v,k\right). \end{array}$$

#### 3.1.1. The Calculation of *f*^*∗*^(*V*(*e*(*v*, *l*), *k*)) and *V*^*∗*^(*e*(*v*, *l*), *k*)

Suppose that, when the phase *j* begins, all values *f*^*∗*^(*V*(*e*(*v*, *s*(*v*))), *k*) have been calculated for each vertex *v* with level lev(*v*) ≥ *L*′ − *j*+1 in *T*. In the phase *j*, we search for all vertices of level *L*′ − *j*. For each of these vertices *v*, we first calculate all values *f*^*∗*^(*e*(*v*, *s*(*v*)), *k*) by the following method, and then, go to the next vertex with level *L*′ − *j*. If *v* is a *G*-vertex or *v*=*h*, we start by assigning

(10) $$\begin{array}{c} W\left(G_{B,v}\right)=w\left(v\right)+\sum_{u\in \text{son}\left(v\right)}W\left(G_{B,u}\right),\\ f^{*}\left(V\left(e\left(v,0\right),1\right)\right)=\sum_{u\in \text{son}\left(v\right)}f^{*}\left(V\left(e\left(u,0\right),1\right)\right)+W\left(G_{B,u}\right)l\left(v,u\right). \end{array}$$

If *v* is a hinge such that *v* ≠ *h*, we start by assigning

(11) $$\begin{array}{c} W\left(G_{B,v}\right)=W\left(G_{v}\right)+\sum_{u\in \text{son}\left(v\right)}W\left(G_{B,u}\right),\\ f^{*}\left(V\left(e\left(v,0\right),1\right)\right)=F_{G_{v}}\left(V\left(G_{v},v,1\right)\right)+\sum_{u\in \text{son}\left(v\right)}f^{*}\left(V\left(e\left(u,0\right),1\right)\right)+W\left(G_{B,u}\right)l\left(v,u\right). \end{array}$$

Assuming that, for all *l*′ < *l*, the values *f*^*∗*^(*V*(*e*(*v*, *l*′), *k*)) have been calculated. Then, the value *f*^*∗*^(*V*(*e*(*v*, *l*), *k*)) can be calculated as follows:

(12) $$\begin{array}{c} f^{*}\left(V\left(e\left(v,l\right),k\right)\right)=\min\left\{\underset{1\leq k^{\prime }\leq k-1}{\min}\left\{f^{*}\left(V\left(e\left(v,l-1\right),k^{\prime }\right)\right)+f^{*}\left(V\left(e\left(v_{l},s\left(v_{l}\right)\right),k-k^{\prime }\right)\right)\right\},\right.\\ \left.f^{*}\left(V\left(e\left(v,l-1\right),k\right)\right)+f^{*}\left(V\left(e\left(v_{l},s\left(v_{l}\right)\right),1\right)\right)+W\left(G_{B,v_{l}}\right)l\left(v,v_{l}\right)\right\}. \end{array}$$

**Figure 2:**
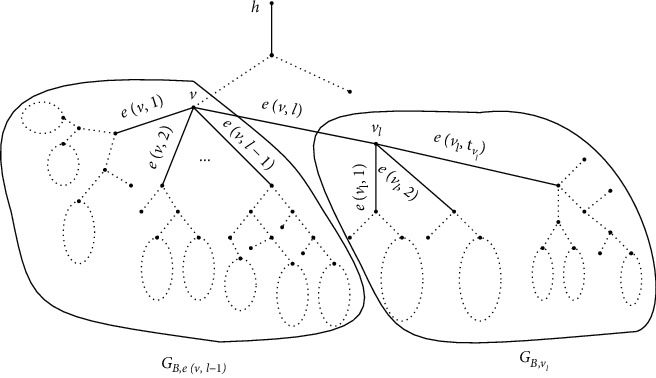
The subgraphs *G*_*B*,*e*(*v*, *l* − 1)_ and *G*_*B*,*v*_*l*__ of *G*_*B*,*v*_.

For the right side of the above formula, the minimal value of the set inside corresponds to the case *v*_*l*_ ∈ *V*^*∗*^(*e*(*v*, *l*), *k*), while the value behind corresponds to the case *v*_*l*_ ∉ *V*^*∗*^(*e*(*v*, *l*), *k*) (Figure 2).

If *v*_*l*_ ∈ *V*^*∗*^(*e*(*v*, *l*), *k*), set

(13) $$\begin{array}{c} V^{*}\left(e\left(v,l\right),k\right)=V^{*}\left(e\left(v,l-1\right),k^{\prime \prime }\right)\cup V^{*}\left(e\left(v_{l},s\left(v_{l}\right)\right),k-k^{\prime \prime }\right), \end{array}$$

where *V*^*∗*^(*e*(*v*, *l* − 1), *k*^″^) and *V*^*∗*^(*e*(*v*_*l*_, *s*(*v*_*l*_)), *k* − *k*^″^) are the corresponding subsets to *f*^*∗*^(*V*(*e*(*v*, *l* − 1), *k*^″^)) and *f*^*∗*^(*V*(*e*(*v*_*l*_, *s*(*v*_*l*_)), *k* − *k*^″^)), respectively. Otherwise, set

(14) $$\begin{array}{c} V^{*}\left(e\left(v,l\right),k\right)=V^{*}\left(e\left(v,l-1\right),k\right). \end{array}$$

Note that there are at most |*V*(*T*)|*p* values *f*^*∗*^(*V*(*e*(*v*, *l*), *k*)) which can be calculated by traversing the edges in *T*. Each calculation contains finding the minimum in at most 2*k* terms. Then, all the values *f*^*∗*^(*V*(*e*(*v*, *l*), *k*)) can be computed in *O*(|*V*(*T*)|*p*^2^) time.

#### 3.1.2. The Calculation of *f*_*G*_*B*,*v*_^*c*^_(*v*)

We start by assigning *f*_*G*_*B*,*h*_^*c*^_(*h*)=0. Suppose that, for all vertices *v* with level lev(*v*) < *j* in *T*, all values *f*_*G*_*B*,*v*_^*c*^_(*v*) have been calculated when the phase *j* begins. In the phase *j*, we traverse all vertices of level *j*. For each of these vertex *v*, we set *W*(*G*_*B*,*v*_^*c*^)=*W*(*G*_*B*,*h*_) − *W*(*G*_*B*,*v*_) and compute *f*_*G*_*B*,*v*_^*c*^_(*v*) as follows:

(15) $$\begin{array}{c} f_{G_{B,v}^{c}}\left(v\right)=f_{G_{B,\text{far}\left(v\right)}^{c}}\left(\text{far}\left(v\right)\right)+f^{*}\left(V\left(e\left(\text{far}\left(v\right),s\left(\text{far}\left(v\right)\right)\right),1\right)\right)-f^{*}\left(V\left(e\left(v,s\left(v\right)\right),1\right)\right)-W\left(G_{B,v}\right)l\left(v,\text{far}\left(v\right)\right)+W\left(G_{B,v}^{c}\right)l\left(v,\text{far}\left(v\right)\right). \end{array}$$

After all values *f*^*∗*^(*V*(*e*(*v*, *l*), *k*)) have been calculated, all values *f*_*G*_*B*,*v*_^*c*^_(*v*) can be calculated by traversing vertices in *T* “downward.” The total calculations take *O*(|*V*(*T*)|) time. Then, all values *F*_*G*_*B*,*h*__(*V*(*G*_*B*,*h*_, *v*, *k*)) can be computed by formula (7) in *O*(|*V*(*T*)|*p*^2^) time.

### 3.2. The Program CYCLE (*B*,*h*)

In this subsection, we are given a cycle *B*_*h*_ and the relevant subcactus *G*_*B*,*h*_; for all pairs {*v*_*i*_, *v*_*j*_} ⊂ *B*_*h*_ and all possible positive numbers *k*, our task is to find all restricted connected *k*-medians *V*(*G*_*B*,*h*_, {*v*_*i*_, *v*_*j*_}, *k*).

Denote by *C* the *B*_*h*_. Assume that *C*{*v*_1_=*h*, *v*_2_,…, *v*_*t*_}, and all vertices are indexed in clockwise. For each vertex *v*_*i*_ in *C*, let *G*_*B*,*v*_1__={*v*_1_}, where *G*_*B*,*v*_*i*__ be the subgraph that contains *v*_*i*_ and all subcacti hanging from it, 2 ≤ *i* ≤ *s*. For any pair *v*_*i*_, *v*_*j*_ ∈ *V*(*C*), *i* ≤ *j*, let *C*_*v*_*i*_,*v*_*j*__(*C*_*v*_*i*_,*v*_*j*__^*co*^) be the subgraph induced by all vertices of the path from *v*_*i*_ to *v*_*j*_ in clockwise (counter-clockwise) direction. Let *G*_*B*,*v*_*i*_,*v*_*j*__(*G*_*B*,*v*_*i*_,*v*_*j*__^*co*^) be the subgraph induced by *C*_*v*_*i*_,*v*_*j*__(*C*_*v*_*i*_,*v*_*j*__^*co*^) and all subcacti hanging from it, and let *G*_*B*,*v*_*i*_,*v*_*j*__^*c*^=*G*_*B*,*v*_1_,*v*_*t*__ − *G*_*v*_*i*_,*v*_*j*__. The calculation of *V*(*G*_*B*,*h*_, {*v*_*i*_, *v*_*j*_}, *k*). Given a pair *v*_*i*_, *v*_*j*_ and a integer *k*, denote by *V*({*v*_*i*_, *v*_*j*_}, *k*) the connected *k*-vertex set in *G*_*B*,*v*_*i*_,*v*_*j*__ that includes *v*_*i*_ and *v*_*j*_. For each integer *k*, 1 ≤ *k* ≤ min{*p*, |*G*_*B*,,*v*_*i*_,*v*_*j*__|}, and the sum of the weighted distances of *V*({*v*_*i*_, *v*_*j*_}, *k*) over *G*_*B*,*v*_*i*_,*v*_*j*__ is defined as

(16) $$\begin{array}{c} f_{1}^{*}\left(V\left(\left\{v_{i},v_{j}\right\},k\right)\right)=\underset{V\left(\left\{v_{i},v_{j}\right\},k\right)\subseteq G_{B,v_{i},v_{j}}}{\min}F_{G_{B,v_{i},v_{j}}}\left(V\left(\left\{v_{i},v_{j}\right\},k\right)\right). \end{array}$$

Let *V*^*∗*^({*v*_*i*_, *v*_*j*_}, *k*) be the corresponding set to *f*_1_^*∗*^(*V*({*v*_*i*_, *v*_*j*_}, *k*)).

Suppose that the midpoint of the path *C*_*v*_*j*_,*v*_*i*__ lies in the edge *e*_*m*(*j*, *i*)_. In particular, if the midpoint is a vertex, assume it coincides with *v*_*m*(*j*, *i*)_. By deleting *e*_*m*(*j*, *i*)_ from *G*_*B*,*v*_*i*_,*v*_*j*__^*c*^, we obtain two subgraphs *G*_*B*,*v*_*i*_,*v*_*j*__^*c*,1^ and *G*_*B*,*v*_*i*_,*v*_*j*__^*c*,2^, which contain *v*_*m*(*j*, *i*)_ and *v*_*m*(*j*, *i*)+1_, respectively. Next, we define

(17) $$\begin{array}{c} f_{2}^{*}\left(V\left(\left\{v_{i},v_{j}\right\},k\right)\right)=\sum_{u\in V\left(G_{B,v_{i},v_{j}}^{c,1}\right)}W\left(G_{B,u}\right)d\left(u,v_{j}\right),\\ f_{3}^{*}\left(V\left(\left\{v_{i},v_{j}\right\},k\right)\right)=\sum_{u\in V\left(G_{v_{i},v_{j}}^{c,2}\right)}W\left(G_{B,u}\right)d\left(u,v_{i}\right), \end{array}$$

to denote the partial sum of the weighted distances to *v*_*j*_ and *v*_*i*_, respectively.

Once all the values defined above have been computed, we can calculate the sum of the weighted distances of *V*(*G*_*B*,*h*_, {*v*_*i*_, *v*_*j*_}, *k*):

(18) $$\begin{array}{c} F_{G_{B,h}}\left(V\left(G_{B,h},\left\{v_{i},v_{j}\right\},k\right)\right)=f_{1}^{*}\left(V\left(\left\{v_{i},v_{j}\right\},k\right)\right)+f_{2}^{*}\left(V\left(\left\{v_{i},v_{j}\right\},k\right)\right)+f_{3}^{*}\left(V\left(\left\{v_{i},v_{j}\right\},k\right)\right). \end{array}$$

We assign *V*(*G*_*B*,*h*_, {*v*_*i*_, *v*_*j*_}, *k*)=*V*^*∗*^({*v*_*i*_, *v*_*j*_}, *k*).

Given a vertex *v*_*i*_ of *C*. If *v*_*i*_ is a *C*-vertex or *v*_*i*_=*h*, we start by assigning

(19) $$\begin{array}{c} W\left(G_{B,v_{i}}\right)=w\left(v_{i}\right),\\ f_{1}^{*}\left(V\left(\left\{v_{i},v_{i}\right\},1\right)\right)=0. \end{array}$$

If *v*_*i*_ is a hinge that is not equal to *h*, we start by assigning

(20) $$\begin{array}{c} W\left(G_{B,v_{i}}\right)=W\left(G_{v_{i}}\right),\\ f_{1}^{*}\left(V\left(\left\{v_{i},v_{i}\right\},1\right)\right)=F_{G_{v_{i}}}\left(V\left(G_{v_{i}},v_{i},1\right)\right). \end{array}$$

Since *C*_*v*_*i*_,*v*_*j*__ is a graft, for all *j* ≥ *i* and possible numbers *k*, the values *f*_1_^*∗*^(*V*({*v*_*i*_, *v*_*j*_}, *k*)) can be calculated by running GRAFT (*B*, *h*) on *G*_*B*,*v*_*i*_,*v*_*j*__. The computation of the values *f*_1_^*∗*^(*V*({*v*_*i*_, *v*_*j*_}, *k*)) for all vertices *v*_*i*_ and *v*_*j*_ of *C* takes *O*(|*V*(*C*)|^2^*p*^2^) time.

For the sake of simplicity, we only describe the calculation of *f*_2_^*∗*^(*V*({*v*_*i*_, *v*_*j*_}, *k*)), while the values of *f*_3_^*∗*^(*V*({*v*_*i*_, *v*_*j*_}, *k*)) can be calculated in a similar way.

Note that, for all pair {*v*_*i*_, *v*_*j*_} of *C*, the corresponding middle edges can be found by the method similar as in [5] in *O*(|*V*(*C*)|^2^) time. For each edge *e*_*m*_=(*v*_*m*_, *v*_*m*+1_) of *C*, denote by *𝒫*(*e*_*m*_)={{*v*_*r*_1__, *v*_*l*_1__}, {*v*_*r*_2__, *v*_*l*_2__},…, {*v*_*r*_*t*__, *v*_*l*_*t*__}} all pairs of vertices of *C* whose middle edge is *e*_*m*_, where *l*_1_ ≥ *l*_2_ ≥ ⋯≥*l*_*t*_. Let *𝒱*={*v*_*l*_1__, *v*_*l*_2__,…, *v*_*l*_*t*__}.

By traversing all vertices in *𝒱*, for *l*_1_ ≥ *l*_*k*_ ≥ *l*_*t*_, all values *f*_2_^*∗*^(*V*({*v*_*r*_*k*__, *v*_*l*_*k*__}, *k*)) can be calculated by the following formula:

(21) $$\begin{array}{c} f_{2}^{*}\left(V\left(\left\{v_{r_{k}},v_{l_{k}}\right\},k\right)\right)=f_{2}^{*}\left(V\left(\left\{v_{r_{k-1}},v_{l_{k-1}}\right\},k\right)\right)+W\left(G_{B,v_{l_{k-1}},v_{m}}\right)d\left(v_{l_{k-1}},v_{l_{k}}\right)+\sum_{l_{k}>j^{\prime }>l_{k-1}}f_{1}^{*}\left(V\left(\left\{v_{j^{\prime }},v_{j^{\prime }}\right\},1\right)\right)+W\left(G_{B,v_{j^{\prime }}}\right)d\left(v_{j^{\prime }},v_{l_{k}}\right), \end{array}$$

in *O*(|*V*(*C*)|) time. Thus, the calculation of all values *f*_2_^*∗*^(*V*({*v*_*i*_, *v*_*j*_}, *k*)) takes *O*(|*V*(*C*)|^2^) time.

Last, the value *W*(*G*_*B*,*h*_) is reassigned as *W*(*G*_*B*,*h*_)=∑_*v*_*i*_∈*B*_*h*__*W*(*G*_*B*,*v*_*i*__), which can be computed in *O*(|*V*(*C*)|) time. Then, all the values *F*_*G*_*B*,*h*__(*V*(*G*_*B*,*h*_, {*v*_*i*_, *v*_*j*_}, *k*)) can be computed by formula (18) in *O*(|*V*(*C*)|^2^*p*^2^) time.

### 3.3. The Program HINGE (*G*_*h*_, *h*)

In this subsection, we are given a hinge *h* and the relevant subcactus *G*_*h*_; for all possible positive integers *k*, our task is to find the restricted connected *k*-medians *V*(*G*_*h*_, *h*, *k*).

**Figure 3:**
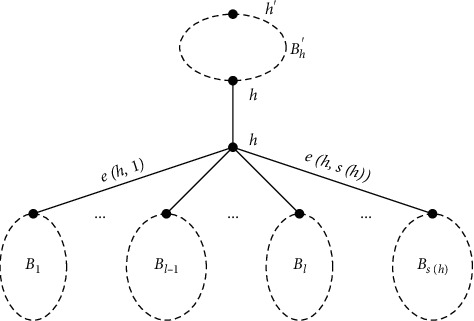
The local structure of *G*_*h*_ in *T*_*G*_.

Assume that *h* is a hinge of *G*. If *h* is the hinge of the block *B*_*i*_ with a lower level in *T*_*G*_, then we call *h* as the farther far(*B*_*i*_) of *B*_*i*_ and *B*_*i*_ as the son of *h*. Let son(*h*) be all sons of *h* and *s*(*h*)=|*E*(*h*)|. Let *E*(*h*) be all edges that incident with *h*. By sorting all edges of *E*(*h*) in an arbitrary order, the *l*^th^ edge is denoted as *e*(*h*, *l*). For each subgraph *G*_*h*_, denote by *G*_*e*(*h*, *l*)_ the maximal connected subgraph that contains *h* and all subcacti hanging from it but not any block *B*_*j*_ for *j* > *l*. Particularly, *G*_*e*(*h*, 0)_=*h* and *G*_*e*(*h*, *s*(*h*))_=*G*_*h*_ (Figure 3).

For each subgraph *G*_*e*(*h*, *l*)_, denote by *V*(*e*(*h*, *l*), *k*) the connected *k*-vertex set that contains *h* but not any vertex of *B*_*j*_ ∈ son(*h*) for *j* > *l*. Next, we define the optimal value of *V*(*e*(*h*, *l*), *k*) over *G*_*e*(*h*, *l*)_.

**Definition 2 .**

In subgraph *G*_*e*(*h*, *l*)_, define

(22) $$\begin{array}{c} f^{*}\left(V\left(e\left(h,l\right),k\right)\right)=\underset{V\left(e\left(h,l\right),k\right)\subseteq G_{e\left(h,l\right)}}{\min}f_{G_{e\left(h,l\right)}}\left(V\left(e\left(h,l\right),k\right)\right), \end{array}$$

where 1 ≤ *k* ≤ min{*p*, |*G*_*e*(*h*, *l*)_|}. Let *V*^*∗*^(*e*(*h*, *l*), *k*) be the corresponding set to *f*^*∗*^(*V*(*e*(*h*, *l*), *k*)).

Once we obtain the values *f*^*∗*^(*e*(*h*, *s*(*h*)), *k*), the sum of weighted distances from vertices of *G*_*h*_ to *V*(*G*_*h*_, *h*, *k*) can be calculated as

(23) $$\begin{array}{c} F_{G_{h}}\left(V\left(G_{h},h,k\right)\right)=f^{*}\left(e\left(h,s\left(h\right)\right),k\right). \end{array}$$

We assign *V*(*G*_*h*_, *h*, *k*)=*V*^*∗*^(*e*(*h*, *s*(*h*)), *k*).

According to our assumption, we obtain

(24) $$\begin{array}{c} f^{*}\left(V\left(e\left(h,0\right),1\right)\right)=\sum_{\text{graft }B_{i}\in \text{son}\left(h\right)}F_{G_{B_{i},h}}\left(V\left(G_{B_{i},h},h,1\right)\right)+\sum_{\text{cycle }B_{j}\in \text{son}\left(h\right)}F_{G_{B_{j},h}}\left(V\left(G_{B_{j},h},\left\{h,h\right\},1\right)\right), \end{array}$$

and assign

(25) $$\begin{array}{c} V^{*}\left(e\left(h,0\right),1\right)=\left\{h\right\}. \end{array}$$

#### 3.3.1. The Calculation of *f*^*∗*^(*V*(*e*(*h*, *l*), *k*)) and *V*^*∗*^(*e*(*h*, *l*), *k*)

Suppose that, when the phase *j* begins, the value *F*_*G*_*B*_*i*_,*h*__(*V*(*G*_*B*_*i*_,*h*_, *h*, 1)) or *F*_*G*_*B*_*i*_,*h*__(*V*(*G*_*B*_*i*_,*h*_, {*h*, *h*}, 1)) has been calculated for each block *B*_*i*_ ∈ *T*_*G*_ of level lev(*B*_*i*_) ≥ *L*_*m*_ − *j*+1. In the phase *j*, we search all hinges of level *L*_*m*_ − *j*. For each of these hinges *h*, we calculate all values *f*^*∗*^(*V*(*e*(*h*, *s*(*h*))), *k*) by the following method, and then, go to the next hinge with level *L*_*m*_ − *j*.

Assuming that, for all *l*′ < *l*, the values *f*^*∗*^(*V*(*e*(*h*, *l*′), *k*)) have been calculated. Then, the value *f*^*∗*^(*V*(*e*(*h*, *l*), *k*)) can be calculated as follows:

(26) $$\begin{array}{c} f^{*}\left(V\left(e\left(h,l\right),k\right)\right)=\underset{1\leq k^{\prime }\leq k-1}{\min}\left\{f^{*}\left(V\left(e\left(h,l-1\right),k^{\prime }\right)\right)+F_{G_{B_{l},h}}\left(V\left(G_{B_{l},h},h,k-k^{\prime }\right)\right)\right\}, \end{array}$$

if *B*_*l*_ is graft, otherwise,

(27) $$\begin{array}{c} f^{*}\left(V\left(e\left(h,l\right),k\right)\right)=\underset{1\leq k^{\prime }\leq k-1}{\min}\left\{f^{*}\left(V\left(e\left(h,l-1\right),k^{\prime }\right)\right)+F_{G_{B_{l},h}}\left(V\left(G_{B_{l},h},\left\{h,h\right\},k-k^{\prime }\right)\right)\right\}. \end{array}$$

If *B*_*l*_ is graft, set

(28) $$\begin{array}{c} V^{*}\left(e\left(h,l\right),k\right)=V^{*}\left(e\left(h,l-1\right),k^{\prime }\right)\cup V\left(G_{B_{l},h},h,k-k^{\prime }\right), \end{array}$$

otherwise, set

(29) $$\begin{array}{c} V^{*}\left(e\left(h,l\right),k\right)=V^{*}\left(e\left(h,l-1\right),k^{\prime }\right)\cup V\left(G_{B_{l},h},\left\{h,h\right\},k-k^{\prime }\right), \end{array}$$

where *V*^*∗*^(*e*(*h*, *l* − 1), *k*′), *V*(*G*_*B*_*l*_,*h*_, *h*, *k* − *k*′), and *V*(*G*_*B*_*l*_,*h*_, {*h*, *h*}, *k* − *k*′) are the corresponding sets to *f*^*∗*^(*V*(*e*(*h*, *l* − 1), *k*′)), *F*_*G*_*B*_*l*_,*h*__(*V*(*G*_*B*_*l*_,*h*_, *h*, *k* − *k*′)), and *F*_*G*_*B*_*l*_,*h*__(*V*(*G*_*B*_*l*_,*h*_, {*h*, *h*}, *k* − *k*′)), respectively.

There are at most |*V*(*T*_*G*_)|*p* values *f*^*∗*^(*V*(*e*(*h*, *l*), *k*)) which can be calculated by traversing the edges in *T*_*G*_. Each calculation contains finding the minimum in at most 2*k* terms. Then, all the values *f*^*∗*^(*V*(*e*(*h*, *l*), *k*)) can be computed in *O*(|*V*(*T*_*G*_)|*p*^2^) time.

Last, *W*(*G*_*h*_) is assigned as *W*(*G*_*h*_)=∑_*B*_*i*_∈son(*h*)_*W*(*G*_*B*_*i*_,*h*_) − *w*(*h*)(*s*(*h*) − 1), which takes constant time.

### 3.4. The Procedure HINGE (*G*_*h*_^*c*^, *h*)

In this subsection, given a hinge *h* and the relevant subcactus *G*_*h*_^*c*^, our task is to calculate the sum of weighted distances from vertices in *G*_*h*_^*c*^s to *h* and the total weights of *G*_*h*_^*c*^.

#### 3.4.1. The Calculation of *F*_*G*_*h*_^*c*^_(*h*) and *W*(*G*_*h*_^*c*^)

We start by assigning *W*(*G*_*B*,*h*_0__^*c*^)=0 and *F*_*G*_*B*,*h*_0__^*c*^_(*h*_0_)=0. Suppose that, when the phase *j* begins, the value of *F*_*G*_*h*_^*c*^_(*h*) and *W*(*G*_*h*_^*c*^) has been calculated for each hinge *h* ∈ *T*_*G*_ with level lev(*h*) < *j*. In the phase *j*, we search all hinges of level *j*. For each of these hinges *h*, set

(30) $$\begin{array}{c} W\left(G_{h}^{c}\right)=W\left(G_{h_{0}}\right)-W\left(G_{h}\right). \end{array}$$

Suppose that far(*h*)=*B*′ in *T*_*G*_ and the hinge of *B*′ is *h*′ (Figure 3). Then, *F*_*G*_*h*_^*c*^_(*h*) can be computed as follows:

(31) $$\begin{array}{c} F_{G_{h}^{c}}\left(h\right)=F_{G_{h^{\prime }}^{c}}\left(h^{\prime }\right)+W\left(G_{h^{\prime }}^{c}\right)d\left(h^{\prime },h\right)+F_{G_{B^{\prime },h^{\prime }}}\left(V\left(G_{B^{\prime },h^{\prime }},h,1\right)\right)-F_{G_{h}}\left(V\left(G_{h},h,1\right)\right), \end{array}$$

if *B*′ is a graft, otherwise,

(32) $$\begin{array}{c} F_{G_{h}^{c}}\left(h\right)=F_{G_{h^{\prime }}^{c}}\left(h^{\prime }\right)+W\left(G_{h^{\prime }}^{c}\right)d\left(h^{\prime },h\right)+F_{G_{B^{\prime },h^{\prime }}}\left(V\left(G_{B^{\prime },h^{\prime }},\left\{h,h\right\},1\right)\right)-F_{G_{h}}\left(V\left(G_{h},h,1\right)\right). \end{array}$$

Note that all values *F*_*G*_*h*_^*c*^_(*h*) and *W*(*G*_*h*_^*c*^) can be calculated by traversing all vertices of *T*_*G*_ “downward.” The total calculations take *O*(|*V*(*T*_*G*_)|) time.

### 3.5. Algorithm for the CpM Problem

According to Lemma 1, we can find a connected *p* median *V*_*p*_^*∗*^ from *𝒱*_1_ ∪ *𝒱*_2_ ∪ *𝒱*_3_. The sum of weighted distance from vertices of *G* to *X*_*p*_^*∗*^ can be calculated as

(33) $$\begin{array}{c} F_{G}\left(V_{p}^{*}\right)=\min\left\{\underset{V\left(G_{B,h},v,p\right)\in V_{1}}{\min}\left\{F_{G_{B,h}}\left(V\left(G_{B,h},v,p\right)\right)+F_{G_{h}^{c}}\left(h\right)+W\left(G_{h}^{c}\right)d\left(h,v\right)\right\},\right.\\ \underset{V\left(G_{B,h},\left\{v_{i},v_{j}\right\},p\right)\in V_{2}}{\min}\left\{F_{G_{B,h}}\left(V\left(G_{B,h},\left\{v_{i},v_{j}\right\},p\right)\right)+F_{G_{h}^{c}}\left(h\right)+W\left(G_{h}^{c}\right)\min\left\{d\left(h,v_{i}\right),d\left(h,v_{j}\right)\right\}\right\},\\ \left.\underset{V\left(G_{h},h,p\right)\in V_{3}}{\min}\left\{F_{G_{h}}\left(V\left(G_{h},h,p\right)\right)+F_{G_{h}^{c}}\left(h\right)\right\}\right\}. \end{array}$$

Next, we design Algorithm 1 to solve the CpM problem. For a given cactus graph *G*, as a preprocessing of Algorithm 1, first, the distance-matrix of the cactus will be calculated. Then, we construct the rooted skeleton graph of the cactus and calculate maximum level *L*_*m*_. This preprocessing will be finished in *O*(*n*^2^) time.

Since there are at most *O*(*n*^2^) elements in *𝒱*_1_ ∪ *𝒱*_2_ ∪ *𝒱*_3_, the calculation of *F*_*G*_(*V*_*p*_^*∗*^) takes *O*(*n*^2^) time. Then, we can use the following theorem to end this section.

**Theorem 1 .**

On the cactus graph with *n* vertices, the CpM problem can be solved in *O*(*n*^2^*p*^2^) time.

## 4. Concluding Remarks

In this article, we consider the connected *p*-median problem on cactus graph and design an algorithm with time complexity *O*(*n*^2^*p*^2^) for it. In the following, it is meaningful to consider the connected *p*-median problems on other classes of graphs, such as planar graphs, interval graphs, and circular-arc graphs.

## Figures and Tables

**Algorithm 1:**
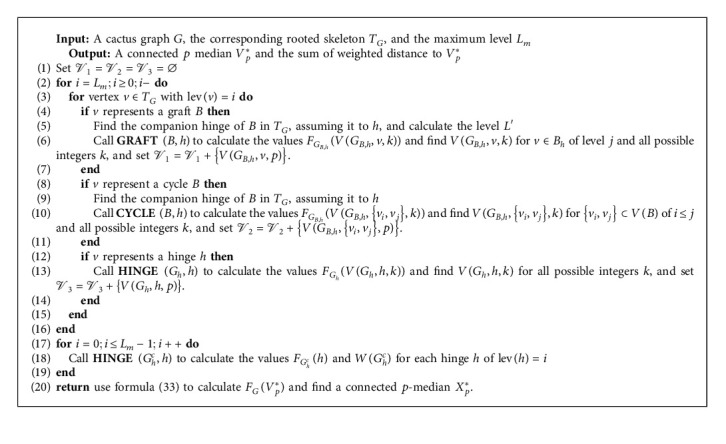
An algorithm for the connected p-median problem of cactus graphs.

## Data Availability

All the datasets used to support the findings of this study are available from the authors upon request.
